# Supplementary material for: Trusting in times of the COVID-19 crisis: Workplace and government trust and depressive symptoms among healthcare workers
Source: Glob Ment Health (Camb). 2025 Oct 13;12:e130. doi: 10.1017/gmh.2025.10067 (PMC12641296; doi:10.1017/gmh.2025.10067)

**Supplementary Materials**

***Moderators***

Human Development Index metric consists of the health dimension (assessed by life expectancy at birth), the education dimension (measured by the mean years of schooling for adults aged 25 years and older), and the standard of living dimension (measured by gross national income per capita; United Nations Development Program, 2019). Values can be categorized into very high human development (≥0.800), high human development (0.700 – 0.799), medium human development (0.550 – 0.699), and low human development (< 0.550).

Stringency Index is a proxy for social distancing during the pandemic, consisting of nine metrics regarding policies during the initial pandemic outbreak: school closures, workplace closures, cancellation of public events, restrictions on public gatherings, closures of public transport, stay-at-home requirements, public information campaigns, restrictions on internal movements, and international travel controls. The index on any given day is calculated as the mean score of the nine metrics, each taking a value between 0 and 100, with a higher score indicating a stricter response (i.e., 100 = strictest response; Hale et al., 2021).

***Covariates***

We used ad-hoc questions to measure the following individual-level variables: age (continuous), gender (0 = “woman”, 1 = “man”, and 2 = “other gender”), educational level, number of household members, current job, and contact with COVID-19 patients (“During the past week, have you been close to patients who were suspected or confirmed cases of COVID-19?”).

To control for the differences in the pandemic severity across the countries we used the infection-per-capita (IPC) and infection-fatality rates (IFR) from paper by Bollyky and colleagues (2022). We obtained these country-level estimates for each of the 22 participating countries at the time when the first participant filled out the survey in each country.

IPC measure indicates the total number of infections per population size, standardized for environmental, demographic, biological, and economic factors such as the relative risk of pneumonia, population density, gross domestic product per capita, the proportion of the population living below 100-meter altitude, and a proxy for previous exposure to other beta coronaviruses (Bollyky, 2022).

IFR measure indicates the risk of death per infection, standardized for environmental, demographic, biological, and economic factors such as the age distribution of the population, mean body-mass index, exposure to air pollution, smoking rates, proxy for previous exposure to other beta coronaviruses, population density, the age-standardized prevalence of chronic obstructive pulmonary disease and cancer, and gross domestic product per capita (Bollyky, 2022).

**Supplementary Table 1**

*Demographic and Trust Variables by Complete and Non-Complete Cases*

|  | Total | Completers n (%) | Non-Completers  n (%) | χ^2^ / t *^c^* | p-value |
| --- | --- | --- | --- | --- | --- |
|  | N = 32,410 | n = 24,782 | n = 7628 |  |  |
| Age, M (SD) | 39.87 (11.22) | 40.22 (11.19) | 38.53 (11.21) | t = -10.15 | <.001 |
| Gender, n (%) |  |  |  | χ^2^ = 14.41 | <.001 |
| Woman | 23,167 (71.5) | 18,255 (73.7) ^a^ | 4,912 (64.4) ^b^ |  |  |
| Man | 8,140 (25.1) | 6,483 (26.2) ^a^ | 1,657 (21.7) |  |  |
| Other | 58 (0.2) | 35 (0.1) ^a^ | 23 (0.3) ^a^ |  |  |
| Education Level, n (%) |  |  |  | χ^2^ = 15.80 | .007 |
| Primary school (not finished) | 53 (0.2) | 32 (0.1) ^a^ | 21 (0.3) ^a^ |  |  |
| Primary school | 212 (0.7) | 170 (0.7) ^a^ | 42 (0.6) ^b^ |  |  |
| Secondary school | 1,958 (6) | 1,557 (6.3) ^a^ | 401 (5.3) ^b^ |  |  |
| Technical-professional | 4,461 (13.8) | 3,456 (14) ^a^ | 996 (13.1) ^b^ |  |  |
| Undergraduate | 11,183 (34.5) | 8,841 (35.7) ^a^ | 2,342 (30.7) ^b^ |  |  |
| Postgraduate | 13,397 (41.3) | 10,698 (43.2) ^a^ | 2,699 (35.4) ^b^ |  |  |
| Members of the Same Household, M(SD) | 3.45 (1.75) | 3.41 (1.71) | 3.60 (1.89) | t = 7.44 | <.001 |
| Facility type, n (%) |  |  |  | χ^2^ = 21.96 | <.001 |
| Non-hospital health center | 8,416 (26) | 6,986 (28.2) ^a^ | 1,430 (18.7) ^b^ |  |  |
| Hospital | 17,772 (54.8) | 14,603(58.9) ^a^ | 3,169 (41.5) ^b^ |  |  |
| Administrative unit outside of a health center | 1,110 (3.4) | 874 (3.5) ^a^ | 236 (3.1) ^b^ |  |  |
| Other care units or centers outside of a health center | 2,823 (8.7) | 2,257 (9.1) ^a^ | 566 (7.4) ^b^ |  |  |
| Current job, n (%) |  |  |  | χ^2^ = 176.48 | <.001 |
| Physician | 8,869 (27.4) | 7,356 (29.7) ^a^ | 1,513 (19.8) ^b^ |  |  |
| Nurse | 6,568 (20.3) | 5,268 (21.3) ^a^ | 1,300 (17) ^b^ |  |  |
| Health Technicians | 3,130 (9.7) | 2,516 (10.2) ^a^ | 614 (8) ^b^ |  |  |
| Ancillary HCWs ^d^ | 3,989 (12.3) | 3,430 (13.8) ^a^ | 559 (7.3) ^b^ |  |  |
| Other HCWs ^e^ | 7,407 (22.5) | 6,173 (24.9) ^a^ | 1,234 (16.2) ^b^ |  |  |
| Trust in the Workplace, M (SD) |  |  |  | χ^2^ = 6.58 | 0.159 |
| Not at all | 2,652 (8.2) | 2,323 (9.4) ^a^ | 329 (4.3) ^b^ |  |  |
| Slightly | 5,682 (17.5) | 4,903 (19.8) ^a^ | 782 (10.3) ^b^ |  |  |
| Moderately | 9,295 (28.7) | 8,104 (32.7) ^a^ | 1,191 (15.6) ^b^ |  |  |
| Considerably | 8,769 (27.1) | 7,669 (30.9) ^a^ | 1,100 (14.4) ^b^ |  |  |
| Extremely | 2,064 (6.4) | 1,775 (7.2) ^a^ | 289 (3.8) ^b^ |  |  |
| Trust in the Government, M (SD) |  |  |  | χ^2^ = 14.483 | 0.006 |
| Not at all | 6,403 (19.8) | 5,512 (22.2) ^a^ | 891 (11.7) ^b^ |  |  |
| Slightly | 8,372 (25.8) | 7,319 (29.5) ^a^ | 1,053 (13.8) ^b^ |  |  |
| Moderately | 8,936 (27.6) | 7,839 (31.6) ^a^ | 1,097 (14.4) ^b^ |  |  |
| Considerably | 4,007 (12.4) | 3,494 (14.1) ^a^ | 513 (6.7) ^b^ |  |  |
| Extremely | 716 (2.2) | 605 (2.4) ^a^ | 111 (1.5) ^b^ |  |  |

***Note.*** Completers had all data on the depressive symptoms (Personal Health Questionnaire-PHQ-9).

Values not sharing the same subscript (^a, b^) are significantly different.

^c^ Value of the Chi square or ^b^ T-test, respectively.

^d^ Ancillary HCWs: e.g., non-clinical manager, administrator/secretary/admission, patient transportation, food/hospitality, cleaning staff, maintenance staff, security staff, student, statistician, analyst, IT, health information management

^e^ Other HCWs: e.g., clinical manager, psychologist, social worker, physical therapist, respiratory therapist, speech therapist, occupational therapist, first responder, midwife, dentist, dentist assistant, dietician, doctor assistant, epidemiologist/public health, pharmacist, community worker, primary attention worker, health promotion/prevention, health educator.

Exact *p*-values are reported unless <.001, in which case “<.001” is shown for readability. Asterisks indicate statistical significance: *p* < .05 (*), *p* < .001 (**).

**Supplementary Table 2**

*Trust in the Workplace and Government, Human Development Index, Stringency Index, Infection per Capita, and Infection Fatality Ratio Stratified by Country*

| Country | All  n (%) |  | Trust in the Workplace | | Trust in the Government | |  | HDI ^a^ | SI ^b^ | IPC | IFR |
| --- | --- | --- | --- | --- | --- | --- | --- | --- | --- | --- | --- |
|  |  |  | Low | High | Low | High |  |  |  |  |  |
| Argentina | 1,038 (3.2) |  | 272 (28.9) | 669 (71.1) | 387 (41.2) | 552 (58.8) |  | 0.851 (Very high) | 61.09 | 248 | 2.95 |
| Armenia | 570 (1.8) |  | 104 (23.9) | 331 (76.1) | 134 (30.8) | 301 (69.2) |  | 0.761 (High) | - | 144 | 2.28 |
| Belgium | 327 (1) |  | 42 (13) | 282 (87) | 188 (58) | 136 (42) |  | 0.939 (Very high) | 36,35 | 188 | 5.4 |
| Bolivia | 167 (0.5) |  | 43 (30.7) | 97 (69.3) | 89 (64) | 50 (36) |  | 0.697 (Medium) | 38,57 | 196 | 9.79 |
| Brazil | 3,246 (10) |  | 511 (17.7) | 2,380 (82.3) | 1,749 (60.5) | 1,141 (39.5) |  | 0.770 (High) | 51,66 | 220 | 2.11 |
| Chile | 2,495 (7.7) |  | 384 (16.6) | 1,926 (83.4) | 1,465 (63.4) | 844 (36.6) |  | 0.856 (Very high) | 70,61 | 132 | 5.84 |
| Colombia | 901 (2.8) |  | 144 (16.2) | 744 (83.8) | 308 (35) | 572 (65) |  | 0.764 (High) | 49,16^c^ | 150 | 2.85 |
| Czech Republic | 1,801 (5.6) |  | 239 (14.6) | 1,403 (85.4) | 499 (30.4) | 1,144 (69.6) |  | 0.898 (Very high) | 41,87 | 80 | 0.81 |
| Germany | 204 (0.6) |  | 20 (10.7) | 167 (89.3) | 27 (14.4) | 160 (85.6) |  | 0.955 (Very high) | 45,68 | 27 | 4.81 |
| Guatemala | 1,813 (5.6) |  | 551 (33.5) | 1,095 (66.5) | 1,234 (75.1) | 409 (24.9) |  | 0.645 (Medium) | 55,15 | 158 | 4.14 |
| Italy | 5,502 (17) |  | 1,710 (34.7) | 3,213 (65.3) | 2,031 (41.3) | 2,889 (58.7) |  | 0.899 (Very high) | 67,02 | 65 | 7.61 |
| Japan | 810 (2.5) |  | 136 (20.8) | 519 (79.2) | 253 (38.6) | 402 (61.4) |  | 0.922 (Very high) | 48,76 | 7 | 9.08 |
| Lebanon | 768 (2.4) |  | 54 (9) | 545 (91) | 405 (67.6) | 194 (32.4) |  | 0.750 (High) | 49,81 | 121 | 2.94 |
| Mexico | 3,253 (10) |  | 1,460 (49.9) | 1,464 (50.1) | 1,921 (65.8) | 1,000 (34.2) |  | 0.763 (High) | 46,80 | 269 | 4.91 |
| Netherlands | 683 (2.1) |  | 66 (10.7) | 548 (89.3) | 76 (12.4) | 538 (87.6) |  | 0.945 (Very high) | 48,82 | 118 | 6.52 |
| Nigeria | 459 (1.4) |  | 194 (50.5) | 190 (49.5) | 154 (40.2) | 229 (59.8) |  | 0.547 (Low) | 43,84 | 144 | 2.42 |
| Peru | 3,670 (11.3) |  | 1,029 (34.6) | 1,947 (65.4) | 1,535 (51.7) | 1,436 (48.3) |  | 0.769 (High) | 67,08 | 310 | 8.13 |
| Puerto Rico | 265 (0.8) |  | 51 (21.2) | 190 (78.8) | 180 (74.7) | 61 (25.3) |  | / | 67,87^d^ | 67 | 1.79 |
| Saudi Arabia | 247 (0.8) |  | 34 (18.3) | 152 (81.7) | 11 (5.9) | 175 (94.1) |  | 0.875 (Very high) | 54,53 | 274 | 5.69 |
| Spain | 2,520 (7.8) |  | 456 (21.1) | 1,707 (78.9) | 1,173 (54.3) | 989 (45.7) |  | 0.901 (Very high) | 49,63 | 107 | 7.66 |
| Tunisia | 633 (2) |  | 334 (58.9) | 233 (41.1) | 440 (77.6) | 127 (22.4) |  | 0.733 (High) | 37,53 | 111 | 1.51 |
| Venezuela | 1,038 (3.2) |  | 503 (60.7) | 326 (39.3) | 516 (62.5) | 310 (37.5) |  | 0.699 (Medium) | 69,03 | 160 | 3.77 |
| Total | 32,410 (100) |  | 8,337 (29.3) | 20,128 (70.7) | 14,775 (52) | 13,659 (48) |  | / | / | / | / |

***Note.*** HDI – Human Development Index, SI – Stringency Index, IPC – Infection per Capita, IFR – Infection Fatality Ratio.

^a^ composite index measuring average achievement in three basic dimensions of human development – a long and healthy life, knowledge, and a decent standard of living. Very high human development (≥0.800), high human development (0.700 – 0.799), medium human development (0.550 – 0.699), and low human development (< 0.550).

^b^ the index records the strictness of ‘lockdown style’ policies that primarily restrict people’s behavior. It is calculated using all ordinal containment and closure policy indicators, plus an indicator recording public information campaigns.

^c^ The average start date of the HEROES survey across countries was June 22, 2020, and the average end date was October 29, 2020. We used these to calculate average SI for Columbia where start and end date of HEROES survey were unavailable.

^d^ We used the average SI of USA National Total score SI to calculate the Puerto Rico SI.

# **Supplementary Table 3**

*Country-level distribution of depressive symptoms (DPHQ) across the HEROES study sample. Percentages refer to the proportion of participants with high depressive symptoms (DPHQ ≥ 10) within each country.*

| **Country** | **N Total** | **High Depressive Symptoms (N)** | **% with**  **High Depressive Symptoms** |
| --- | --- | --- | --- |
| Chile | 2495 | 966 | 38.7% |
| Tunisia | 633 | 209 | 33.0% |
| Brazil | 3246 | 1009 | 31.1% |
| Bolivia | 167 | 50 | 29.9% |
| Spain | 2520 | 721 | 28.6% |
| Argentina | 1038 | 272 | 26.2% |
| Armenia | 570 | 149 | 26.1% |
| Italy | 5502 | 1435 | 26.1% |
| Guatemala | 1813 | 467 | 25.8% |
| Saudi Arabian | 247 | 61 | 24.7% |
| Mexico | 3253 | 730 | 22.4% |
| Peru | 3670 | 799 | 21.8% |
| Colombia | 901 | 191 | 21.2% |
| Puerto Rico | 265 | 54 | 20.4% |
| Lebanon | 768 | 154 | 20.1% |
| Germany | 204 | 35 | 17.2% |
| Venezuela | 1038 | 175 | 16.9% |
| Belgium | 327 | 53 | 16.2% |
| Czech Republic | 1801 | 244 | 13.5% |
| Japan | 810 | 80 | 9.9% |
| Netherlands | 683 | 63 | 9.2% |
| Nigeria | 459 | 42 | 9.2% |
| **Total** | **32410** | **7959** | **24.6%** |

# **Supplementary Table 4**

*Distribution of Trust in Government and Workplace by Country*

| **Country** | **Gov. 0** | **Gov. 1** | **Gov. 2** | **Gov. 3** | **Gov. 4** | **Work. 0** | **Work. 1** | **Work. 2** | **Work. 3** | **Work. 4** | **Total N** |
| --- | --- | --- | --- | --- | --- | --- | --- | --- | --- | --- | --- |
| **Argentina** | 150 | 288 | 278 | 265 | 57 | 89 | 210 | 304 | 361 | 74 | 1038 |
| **Armenia** | 100 | 99 | 200 | 116 | 55 | 55 | 89 | 174 | 177 | 75 | 570 |
| **Belgium** | 66 | 125 | 99 | 37 | 0 | 13 | 31 | 122 | 120 | 41 | 327 |
| **Bolivia** | 50 | 59 | 44 | 11 | 3 | 14 | 41 | 52 | 52 | 8 | 167 |
| **Brazil** | 832 | 1146 | 852 | 340 | 76 | 106 | 501 | 1211 | 1134 | 294 | 3246 |
| **Chile** | 843 | 739 | 610 | 268 | 35 | 100 | 327 | 743 | 1093 | 232 | 2495 |
| **Colombia** | 99 | 223 | 379 | 160 | 40 | 21 | 125 | 330 | 319 | 106 | 901 |
| **Czech Republic** | 176 | 393 | 829 | 377 | 26 | 57 | 214 | 661 | 785 | 84 | 1801 |
| **Germany** | 10 | 22 | 75 | 84 | 13 | 4 | 21 | 54 | 79 | 46 | 204 |
| **Guatemala** | 761 | 593 | 304 | 124 | 31 | 187 | 420 | 456 | 578 | 172 | 1813 |
| **Italy** | 589 | 1682 | 2396 | 740 | 95 | 521 | 1401 | 2249 | 1076 | 255 | 5502 |
| **Japan** | 77 | 244 | 303 | 182 | 4 | 28 | 145 | 266 | 324 | 47 | 810 |
| **Lebanon** | 283 | 246 | 199 | 31 | 9 | 17 | 63 | 208 | 298 | 182 | 768 |
| **Mexico** | 1032 | 1109 | 746 | 306 | 60 | 630 | 983 | 843 | 670 | 127 | 3253 |
| **Netherlands** | 28 | 69 | 313 | 248 | 25 | 7 | 74 | 227 | 304 | 71 | 683 |
| **Nigeria** | 52 | 131 | 155 | 91 | 30 | 119 | 107 | 121 | 89 | 23 | 459 |
| **Peru** | 707 | 1217 | 1199 | 484 | 63 | 335 | 916 | 1194 | 1042 | 183 | 3670 |
| **Puerto Rico** | 115 | 80 | 56 | 8 | 6 | 17 | 38 | 69 | 100 | 41 | 265 |
| **Saudi Arabian** | 6 | 18 | 42 | 100 | 81 | 18 | 30 | 72 | 90 | 37 | 247 |
| **Spain** | 737 | 655 | 708 | 381 | 39 | 163 | 412 | 793 | 1011 | 141 | 2520 |
| **Tunisia** | 248 | 235 | 127 | 21 | 2 | 178 | 180 | 168 | 90 | 17 | 633 |
| **Venezuela** | 366 | 289 | 222 | 116 | 45 | 296 | 299 | 230 | 165 | 48 | 1038 |

***Note.*** Values represent the absolute number of respondents within each country who endorsed a given level of trust in government (Gov. 0 to Gov. 4) and trust in workplace (Work. 0 to Work. 4). Trust levels are based on a 5-point Likert scale: 0 = Not at all, 1 = Slightly, 2 = Moderately, 3 = Considerably, 4 = Extremely. Total_N reflects the number of respondents per country with available data on both trust variables. Percentages are not shown here but are visualized in *Supplementary Figures 1* and *2*.

#### **Supplementary Figure 1** Heatmap of Trust in Government by Country

**
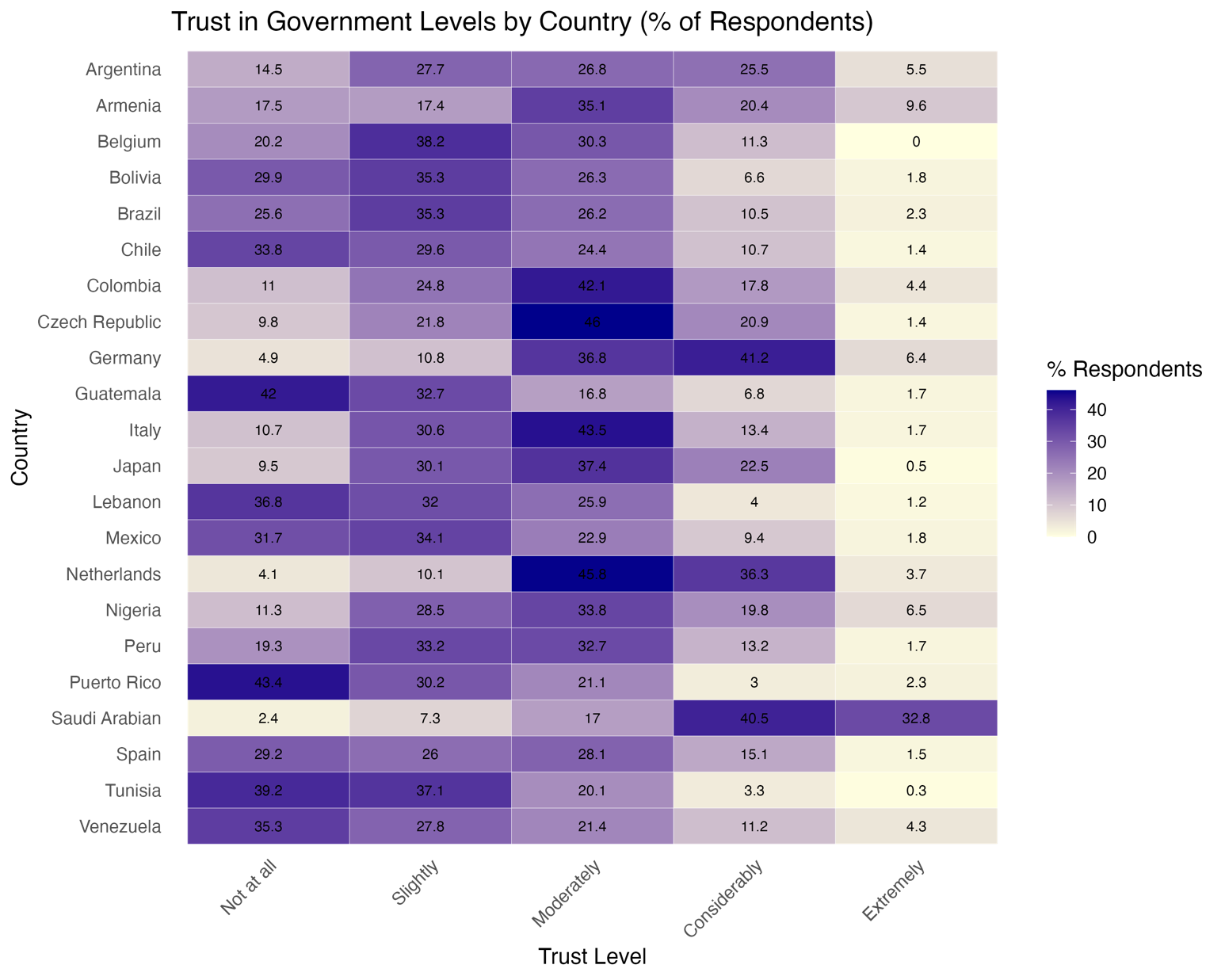
**

***Note.*** Each cell indicates the percentage of respondents in a given country who selected that level of trust in government (0 = Not at all; 4 = Extremely). Rows represent countries; columns represent trust levels. Darker colors represent higher proportions.

#### **Supplementary Figure 2** Heatmap of Trust in Workplace by Country

**
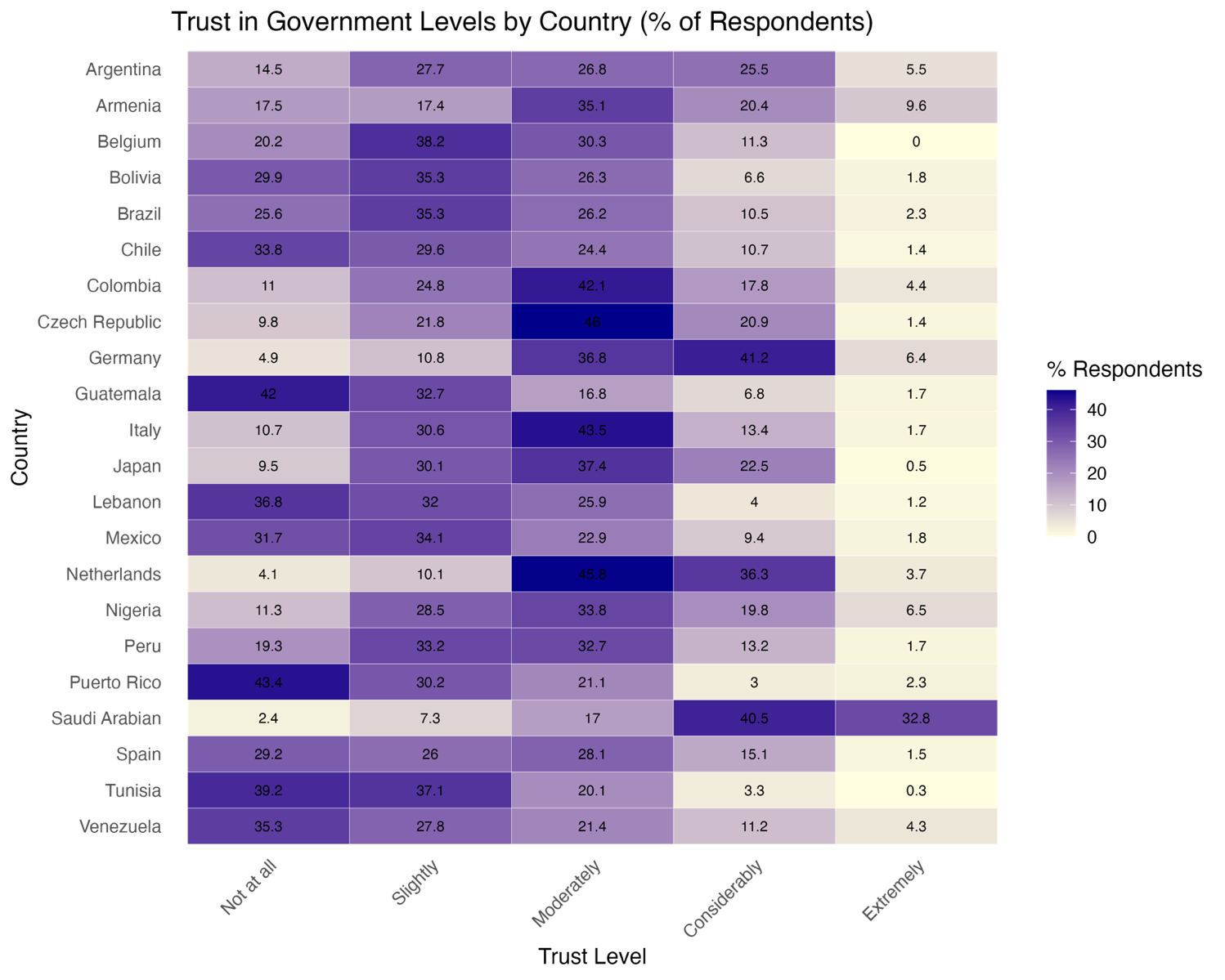
**

*Note.* Each cell indicates the percentage of respondents in a given country who selected that level of trust in their workplace (0 = Not at all; 4 = Extremely). Rows represent countries; columns represent trust levels. Warmer colors represent higher proportions.

**Supplementary Table 5**

*Multilevel Model for Depressive Symptoms*

| **Variables** | **β** | **SE** | **Lower CI** | **Upper CI** |
| --- | --- | --- | --- | --- |
| ***Fixed Effects*** |  |  |  |  |
| **Individual Level** | | | | |
| Trust in the Workplace ^a^ |  |  |  |  |
| Slightly | -.618* | .115 | -.843 | -.393 |
| Moderately | -1.202** | .112 | -1.421 | -.983 |
| Considerably | -1.827** | .115 | -2.052 | -1.602 |
| Extremely | -2.344** | .151 | -2.639 | -2.049 |
| Trust in the Government ^f^ |  |  |  |  |
| Slightly | -.871** | .081 | -1.030 | -.712 |
| Moderately | -1.375** | .084 | -1.540 | -1.210 |
| Considerably | -1.788** | .105 | -1.994 | -1.582 |
| Extremely | -1.537** | .205 | -1.938 | -1.136 |
| **Country Level** | | | | |
| Stringency Index | .041 | .028 | -.014 | .094 |
| Human Development Index ^b^ |  |  |  |  |
| Medium | 1.659 | 1.587 | -1.451 | 4.769 |
| High | 1.844 | 1.435 | -0.969 | 4.657 |
| Very High | 2.201 | 1.446 | -0.633 | 5.035 |
| Infection-per-Capita | .004 | .004 | -.004 | .012 |
| Infection-fatality-Ratio | -.069 | .121 | -.306 | .168 |
| ***Random effects*** |  |  |  |  |
| Country variation | 1.643 | 1.282 | 0.961 | 1.810 |
| Individual variation | 25.508 | 5.051 | 5.012 | 5.090 |

***Note*.**

^a^ Reference category is “not at all”.

^b^ Reference category is low HDI.

Exact *p*-values are reported unless <.001, in which case “<.001” is shown for readability. Asterisks indicate statistical significance: *p* < .05 (*), *p* < .001 (**).

**Supplementary Table 6**

*Multilevel Model for Depressive Symptoms (Covariates - Only)*

| **Variables** | **β** | **SE** | **Lower CI** | **Upper CI** |
| --- | --- | --- | --- | --- |
| ***Fixed Effects*** |  |  |  |  |
| **Individual Level** | | | | |
| Age | -.040** | .003 | -.047 | -.034 |
| Gender ^a^ |  |  |  |  |
| Man | -.932** | .084 | -1.097 | -.766 |
| Other | 2.449** | .766 | .947 | 3.950 |
| Education ^b^ |  |  |  |  |
| Primary school | -1.369 | .956 | -3.244 | .505 |
| Secondary school | -.551 | .866 | -2.248 | 1.146 |
| Technical-professional training | -.719 | .861 | -2.407 | .970 |
| Undergraduate studies | -.568 | .859 | -2.251 | 1.114 |
| Postgraduate studies | -.810 | .859 | -2.494 | .873 |
| Number of Household Members | -.060* | .021 | -.103 | -.017 |
| Current job ^c^ |  |  |  |  |
| Physician | .315** | .105 | .109 | .521 |
| Nurse | .554** | .108 | .341 | .767 |
| Health technician | .262 | .139 | -.012 | .535 |
| Ancillary HCW | .044 | .125 | -.201 | .289 |
| Contact with COVID-19 patients ^d^ | .779** | .081 | .621 | .938 |
| **Country Level** | | | | |
| Stringency Index | 0.041 | 0.028 | -0.018 | 0.101 |
| Human Development Index ^e^ |  |  |  |  |
| Medium | 1.659 | 1.587 | -1.943 | 5.261 |
| High | 1.844 | 1.435 | -1.016 | 4.704 |
| Very High | 2.201 | 1.446 | -0.758 | 5.161 |
| Infection-per-Capita | 0.004 | 0.004 | -0.004 | 0.011 |
| Infection-fatality-Ratio | -0.069 | 0.121 | -0.305 | 0.167 |
| ***Random effects*** |  |  |  |  |
| Country variation | 1.643 | 1.282 | 0.961 | 1.810 |
| Individual variation | 25.508 | 5.051 | 5.012 | 5.090 |

***Note*.**

^a^ Reference category is woman.

^b^ Reference category is incomplete primary education.

^c^ Reference category is other HCWs.

^d^ Reference category is no contact with COVID-19 patients.

^e^ Reference category is low HDI.

Exact *p*-values are reported unless <.001, in which case “<.001” is shown for readability. Asterisks indicate statistical significance: *p* < .05 (*), *p* < .001 (**).

**Supplementary Table 7**

*Model Fit Comparisons Between Random Intercept and Random Slope Multilevel Models Predicting PHQ-9 and GHQ-12 Scores From Trust in Workplace and Government*

| **Outcome** | **Predictor** | **Model Type** | **AIC** | **BIC** | **logLik** | **Δχ²** | **df** | **p-value** |
| --- | --- | --- | --- | --- | --- | --- | --- | --- |
| PHQ-9 | Trust Workplace | Intercept-only | 193649.8 | 193792.1 | -96807.9 | — | — | — |
|  |  | Random slope | 193561.6 | 193720.8 | -96761.8 | 91.8 | 2 | < .001 |
| PHQ-9 | Trust Government | Intercept-only | 193649.8 | 193792.1 | -96807.9 | — |  |  |
|  |  | Random slope | 193640.3 | 193799.3 | -96801.2 | 13.5 | 2 | < .001 |

**Note.** Δχ² statistics reflect the difference in -2 log-likelihood between random intercept and random slope models for each outcome and trust predictor. All comparisons were made using maximum likelihood estimation (ML). Random slope models allow the effect of trust to vary across countries. Lower AIC and BIC values indicate better model fit.

Exact *p*-values are reported unless <.001, in which case “<.001” is shown for readability. Asterisks indicate statistical significance: *p* < .05 (*), *p* < .001 (**).

### **Supplementary Table 8**

### *Moderation of the Association Between Trust and Depressive Symptoms by Country-Level Indicators (HDI, SI)*

| **Outcome** | **Predictor × Moderator** | **B** | **SE** | **95% CI** | **p-value** |
| --- | --- | --- | --- | --- | --- |
| PHQ-9 | Trust Workplace × HDI | –1.718 | 0.304 | [-2.314, -1.122] | <.001 |
| PHQ-9 | Trust Workplace × SI | -0.009 | 0.003 | [-0.015, -0.004] | <.001 |
| PHQ-9 | Trust Government × HDI | -0.030 | 0.324 | [-0.666, 0.606] | .927 |
| PHQ-9 | Trust Government × SI | 0.003 | 0.003 | [-0.002, 0.009] | .249 |

**Note.** Models included age, gender, education, household size, occupational role, and patient contact (individual-level covariates), as well as HDI, SI, Infection-Per-Capita (IPC), and Infection-Fatality Ratio (IFR) (country-level covariates). SI = Stringency Index. HDI = Human Development Index. PHQ-9 = Patient Health Questionnaire (Depressive Symptoms).

Exact *p*-values are reported unless <.001, in which case “<.001” is shown for readability. Asterisks indicate statistical significance: *p* < .05 (*), *p* < .001 (**).

**Supplementary Table 9**

*Proportion of Missing Data per Variable Included in the Analyses (N = 32,410)*

| **Variable** | **Missing Percent** |
| --- | --- |
| Mental Illness (prepandemic diagnosis) | 37.24% |
| Physical Illness (prepandemic diagnosis) | 36.03% |
| Exposure (contact with COVID-19 patients) | 25.50% |
| Depresive symptoms (PHQ-9) | 23.12% |
| Age | 15.08% |
| Trust Government | 12.27% |
| Trust Workpalce | 12.17% |
| Patient Population | 9.44% |
| Sector (public or private) | 8.32% |
| Current Job | 7.55% |
| Workplace | 6.86% |
| Number of Household Members | 4.08% |
| Eduaction | 3.54% |
| Gender | 3.22% |
| ID | 0 |
| Country | 0 |

# **Supplementary Table 10**

# *Logistic Regression Models Using Continuous Trust Variables to Predict Depressive Symptoms (DPHQ)*

| **Predictor** | **Estimate (log odds)** | **SE** | **z value** | **OR [95% CI]** | **p-value** |
| --- | --- | --- | --- | --- | --- |
| Intercept | -1.510 | 0.117 | -12.87 | 0.22 [0.18, 0.26] | < .001 |
| Trust in Workplace (z) | -0.279 | 0.014 | -19.92 | 0.76 [0.74, 0.78] | < .001 |
| Age (z) | -0.211 | 0.018 | -12.04 | 0.81 [0.78, 0.83] | < .001 |
| Gender (female) | -0.311 | 0.032 | -9.79 | 0.73 [0.69, 0.77] | < .001 |
| Education (z) | -0.030 | 0.014 | -2.18 | 0.97 [0.94, 0.99] | .029 |
| Household Members (z) | -0.028 | 0.014 | -1.96 | 0.97 [0.94, 1.00] | .051 |
| Current Job | -0.002 | 0.002 | -1.05 | 1.00 [0.99, 1.00] | .293 |
| Exposure | 0.410 | 0.030 | 13.44 | 1.51 [1.42, 1.60] | < .001 |
|  |  |  |  |  |  |
| Intercept | -1.489 | 0.113 | -13.15 | 0.23 [0.19, 0.27] | < .001 |
| Trust in Government (z) | -0.260 | 0.014 | -18.09 | 0.77 [0.75, 0.79] | < .001 |
| Age (z) | -0.196 | 0.018 | -11.17 | 0.82 [0.80, 0.85] | < .001 |
| Gender (female) | -0.323 | 0.032 | -10.22 | 0.72 [0.68, 0.76] | < .001 |
| Education (z) | -0.030 | 0.014 | -2.22 | 0.97 [0.94, 0.99] | .027 |
| Household Members (z) | -0.027 | 0.014 | -1.87 | 0.97 [0.94, 1.00] | .062 |
| Current Job | -0.002 | 0.002 | -0.94 | 1.00 [0.99, 1.00] | .346 |
| Exposure | 0.392 | 0.030 | 12.86 | 1.48 [1.40, 1.57] | < .001 |

**Note.** Multilevel logistic regression models with country as a random intercept. Trust in Workplace and Trust in Government were standardized (z-scores). Odds ratios (ORs) and 95% confidence intervals (CIs) are presented. All models adjusted for age, gender, education, number of household members, current job, and exposureto COVID-19 patients.

**Supplementary Figure 3**

*Map of the Countries Participating in the Study*


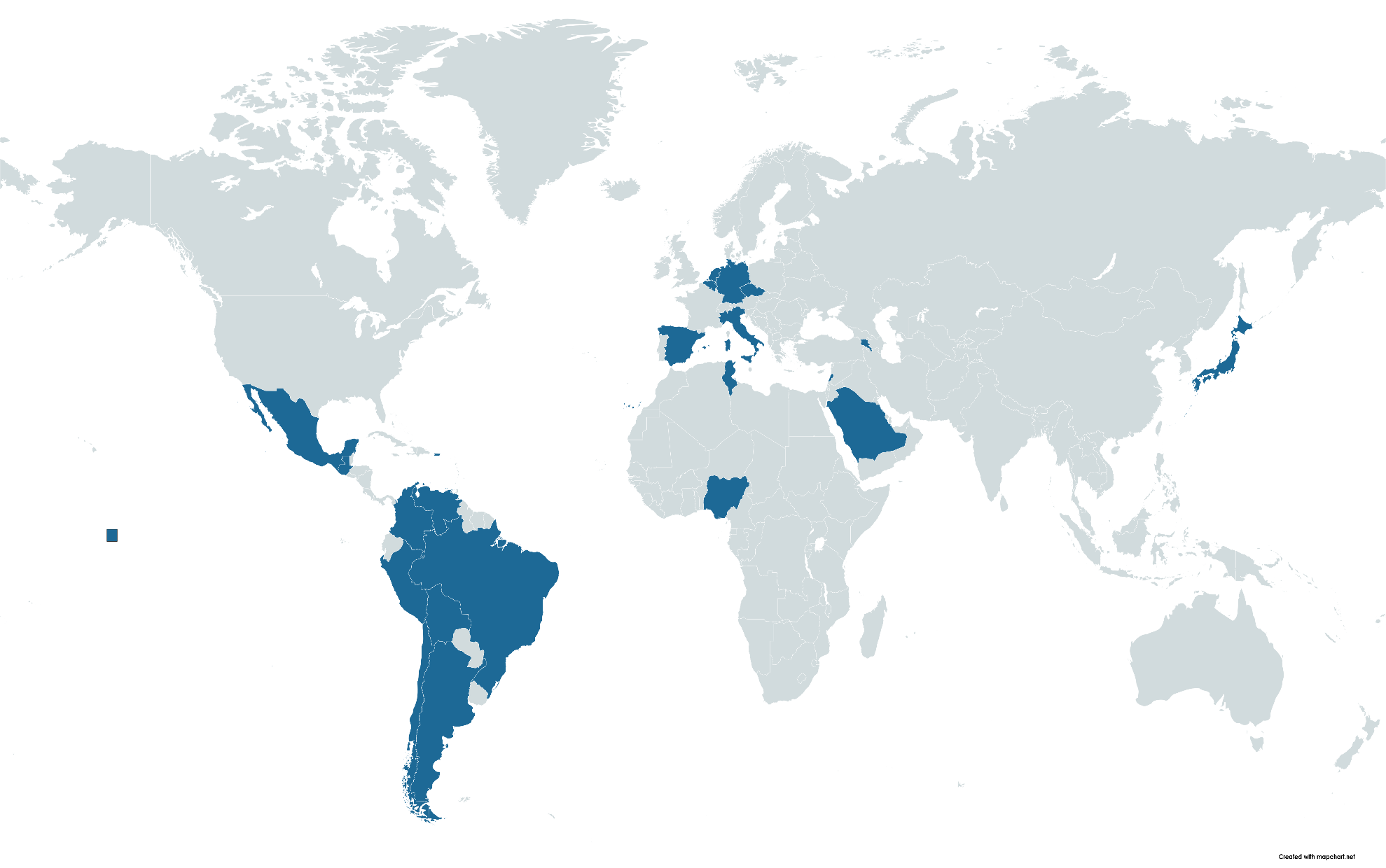

Supplement: Basic et al. supplementary material [file S2054425125100678sup001.docx]
